# CyberKnife^® ^enhanced conventionally fractionated chemoradiation for high grade glioma in close proximity to critical structures

**DOI:** 10.1186/1756-8722-3-22

**Published:** 2010-06-09

**Authors:** Eric Oermann, Brian T Collins, Kelly T Erickson, Xia Yu, Sue Lei, Simeng Suy, Heather N Hanscom, Joy Kim, Hyeon U Park, Andrew Eldabh, Christopher Kalhorn, Kevin McGrail, Deepa Subramaniam, Walter C Jean, Sean P Collins

**Affiliations:** 1 Department of Radiation Oncology, Georgetown University Hospital, Washington, DC, USA; 2 Department of Neurosurgery, Georgetown University Hospital, Washington, DC, USA; 3 Department of Hematology and Oncology, Georgetown University Hospital, Washington, DC, USA

## Abstract

**Introduction:**

With conventional radiation technique alone, it is difficult to deliver radical treatment (≥ 60 Gy) to gliomas that are close to critical structures without incurring the risk of late radiation induced complications. Temozolomide-related improvements in high-grade glioma survival have placed a higher premium on optimal radiation therapy delivery. We investigated the safety and efficacy of utilizing highly conformal and precise CyberKnife radiotherapy to enhance conventional radiotherapy in the treatment of high grade glioma.

**Methods:**

Between January 2002 and January 2009, 24 patients with good performance status and high-grade gliomas in close proximity to critical structures (i.e. eyes, optic nerves, optic chiasm and brainstem) were treated with the CyberKnife. All patients received conventional radiation therapy following tumor resection, with a median dose of 50 Gy (range: 40 - 50.4 Gy). Subsequently, an additional dose of 10 Gy was delivered in 5 successive 2 Gy daily fractions utilizing the CyberKnife^® ^image-guided radiosurgical system. The majority of patients (88%) received concurrent and/or adjuvant Temozolmide.

**Results:**

During CyberKnife treatments, the mean number of radiation beams utilized was 173 and the mean number of verification images was 58. Among the 24 patients, the mean clinical treatment volume was 174 cc, the mean prescription isodose line was 73% and the mean percent target coverage was 94%. At a median follow-up of 23 months for the glioblastoma multiforme cohort, the median survival was 18 months and the two-year survival rate was 37%. At a median follow-up of 63 months for the anaplastic glioma cohort, the median survival has not been reached and the 4-year survival rate was 71%. There have been no severe late complications referable to this radiation regimen in these patients.

**Conclusion:**

We utilized fractionated CyberKnife radiotherapy as an adjunct to conventional radiation to improve the targeting accuracy of high-grade glioma radiation treatment. This technique was safe, effective and allowed for optimal dose-delivery in our patients. The value of image-guided radiation therapy for the treatment of high-grade gliomas deserves further study.

## Introduction

High-grade gliomas are generally aggressive tumors with poor prognosis [1]. They tend to recur locally [2] and rarely spread beyond the confines of the central nervous system. Therefore, local control is considered the primary determinant of overall survival. Treatment routinely consists of maximum safe surgery followed by postoperative conventionally fractionated radiation therapy plus or minus chemotherapy [3-6]. With standard therapy, including Temozomide, the 2 year overall survival estimate for glioblastoma multiforme (GBM) is an improved but yet still disappointing 27% [4]. Anaplastic glioma outcomes are considerably better with a 4 year overall survival estimate of approximately 50% [5,6]. Current practice guidelines recommend treating high-grade gliomas with conventionally fractionated (1.8 - 2.0 Gy) partial brain irradiation over an approximately 6 week period [7]. The gross tumor volume (GTV) is targeted with large margins (2-3 cm) too addresses deep subclinical brain infiltration [8]. Radiosurgy with or without conventional irradiation is not recommended at this time given the poor tolerance of the normal brain to hypofractionation [9] and disappointing published treatment outcomes [10-13].

Presently, it is our clinical practice to treat high-grade glioma patients with maximum safe surgery followed by 6 weeks of chemoradiation (60 Gy partial brain irradiation in 2 Gy fractions with concurrent and adjuvant Temozolomide). It has been generally feasible with conventional radiation technique to deliver such "full dose" treatment while respecting institutional peritumoral critical structure maximum point dose tolerances (Table 1). However, for some deep seated tumors, typically involving the temporal and frontal lobes, such treatment is often not feasible with conventional treatment inaccuracies approaching 5 mm in the best hands [14,15]. Historically, the total radiation dose has been lowered in such cases to protect normal tissue function with the understanding that such treatment modifications could adversely affect overall survival [16]. With recent Temozolomide-related improvements in high-grade glioma survival [4], it is now more likely than ever that suboptimal radiation treatment will result in either a decrement in overall survival or an increase in late radiation toxicity.

**Table 1:** Cumulative Radiation Maximum Point Dose Limits

Critical Structure	Maximum Point Dose Limit (total for 30 fractions)
Lens	10 Gy

Retina	50 Gy

Optic Nerve	55 Gy

Optic Chiasm	55 Gy

*Brainstem*	55 Gy

The CyberKnife^®^, a commercially available frameless image-guided radiosurgery system (Accuray, Sunnyvale, CA), was installed at Georgetown University Hospital in late 2001. Standard components include a light weight linear accelerator, a robotic manipulator and an automated x-ray image-guided computer targeting system. Generally, the treatment planning system with input from the user selects hundreds of small non-isocentric circular radiation beams to deliver a highly conformal radiation treatment with steep dose gradients to a defined target in order to spare normal tissues [17,18]. Subsequently, the automated robotic manipulator directed by the frequently updated x-ray targeting system's knowledge of the patient's unique cranial anatomy efficiently delivers the selected radiation beams with submilimeter accuracy. We report the safety and efficacy of using the highly conformal and accurate CyberKnife radiosurgery system to enhance the final week of conventional radiotherapy in 24 patients with high-grade gliomas in close proximity to critical structures.

## Patients and Methods

### Patient Population

Patients with newly diagnosed resected unifocal high-grade gliomas (WHO Grade III and VI) in close proximity (<1 cm) to critical structures (Table 2) were evaluated. All patients were in RPA class 1 to 4 [19,20]. Magnetic resonance imaging (MRI) was completed preoperatively and postoperatively. The Georgetown University Hospital institutional review board approved this study and all participants provided informed written consent.

**Table 2:** Patient Characteristics

Patient	Histology	Resection	Chemotherapy	Lobe	RPA	Age	Sex	Deficit
1	Glioblastoma multiforme	Total	Concurrent and Adjuvant	Frontal-L	4	60	Male	No

2	Glioblastoma multiforme	Subtotal	Concurrent and Adjuvant	Frontal-L	3	44	Female	No

3	Anaplastic oligodendroglioma	Total	Adjuvant	Frontal-L	1	27	Male	No

4	Anaplastic oligoastrocytoma	Total	None	Frontal-R	1	33	Male	No

5	Anaplastic astrocytoma	Total	Adjuvant	Frontal-R	1	42	Female	No

6	Anaplastic oligodendroglioma	Total	Concurrent and Adjuvant	Frontal-R	1	42	Male	No

7	Anaplastic astrocytoma	Total	Adjuvant	Frontal-R	1	39	Female	No

8	Anaplastic astrocytoma	Subtotal	Concurrent and Adjuvant	Frontal-R	2	62	Female	Yes

9	Glioblastoma multiforme	Total	Concurrent and Adjuvant	Occipital-R	4	70	Female	No

10	Anaplastic oligoastrocytoma	Total	Adjuvant	Parietal-R	1	48	Male	No

11	Anaplastic oligoastrocytoma	Total	Adjuvant	Temporal-L	1	42	Male	No

12	Glioblastoma multiforme	Total	Concurrent and Adjuvant	Temporal-L	4	72	Female	No

13	Anaplastic astrocytoma	Subtotal	Concurrent and Adjuvant	Temporal-L	1	28	Female	No

14	Glioblastoma multiforme	Subtotal	Concurrent and Adjuvant	Temporal-L	4	51	Female	No

15	Anaplastic astrocytoma	Total	Concurrent and Adjuvant	Temporal-R	2	66	Female	No

16	Glioblastoma multiforme	Subtotal	Concurrent and Adjuvant	Temporal-R	4	63	Female	No

17	Glioblastoma multiforme	Subtotal	Concurrent and Adjuvant	Temporal-R	4	59	Female	No

18	Glioblastoma multiforme	Subtotal	Concurrent and Adjuvant	Temporal-R	4	56	Male	No

19	Anaplastic astrocytoma	Subtotal	Concurrent and Adjuvant	Temporal-R	2	67	Male	No

20	Glioblastoma multiforme	Total	Concurrent and Adjuvant	Temporal-R	4	69	Male	No

21	Anaplastic astrocytoma	Total	Concurrent and Adjuvant	Temporal-R	1	16	Male	No

22	Glioblastoma multiforme	Subtotal	Concurrent and Adjuvant	Temporal-R	4	55	Male	No

23	Glioblastoma multiforme	Subtotal	Concurrent and Adjuvant	Temporal-R	4	57	Male	No

24	Glioblastoma multiforme	Subtotal	Concurrent and Adjuvant	Temporal-R	4	65	Female	No

### Surgery

The extent of surgical resection was documented as total tumor resection or subtotal tumor resection following review of operative reports and post operative MRI imaging (Table 2). Salvage surgery was routinely recommended for patients with good performance status and evidence of recurrence or radiation necrosis based on imaging studies.

### Conventional Radiation Treatment

Patients were placed in the supine treatment position with their heads resting on a standard support. A custom thermoplastic mask was crafted. Thin-sliced (1.25 mm) high-resolution CT images were obtained through the cranium for conventional and CyberKnife treatment planning. Treatment planning MRI imaging was completed selectively to enhance target and critical structure delineation when clinically indicated. Target volumes and critical structures were contoured by team neurosurgeons. Treatment volumes were generous including the contrast enhancing tumor volume when present and the surgical defect with a 3 cm margin. Critical structures in close proximity to the target volume were not excluded from the treatment volume during conventional radiation treatment. Forty to 50.4 Gy was delivered in 1.8 to 2.0 Gy fractions 5 days a week for a total of 4 to 5 1/2 weeks. Treatment was delivered using linear accelerators with nominal energies ≥ 6 MV. Intensity modulated radiation therapy (IMRT) technique was not permitted.

### CyberKnife Treatment

**Figure 1:**
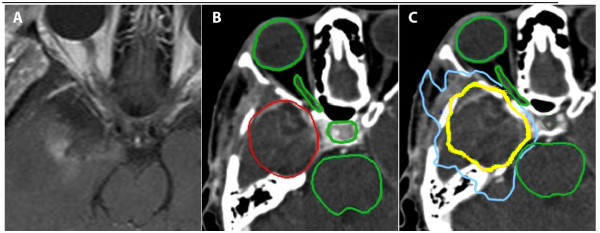
**(A) Axial T1-weighted post contrast MRI illustrating a right-sided temporal lobe high-grade glioma resection cavity bordering the right optic nerve, optic chiasm and brainstem**. **(B) **Planning Axial CT image. The radiosurgical planning treatment volume is contoured in red and critical structures are contoured in green. **(C) **Planning Axial CT illustrating the prescription isodose line in yellow and the 50% isodose line in blue.

Following the completion of conventional radiation therapy, CyberKnife treatment was completed without a planned treatment break (Figure 1). The technical aspects of CyberKnife^® ^radiosurgical system for cranial tumors have been described in detail [17,18]. The treatment volume for the radiosurgical boost included the contrast-enhancing lesion and the resection cavity as defined by the patient's neurosurgeon plus a 1 cm margin when clinically indicated (Figure 1A, B). Due to the submillimeter precision of CyberKnife treatment, no additional margin was added to correct for set-up inaccuracy. The treating neurosurgeon and radiation oncologist in consultation determined the prescription isodose line (Figure 1C). Twelve circular collimator ranging in diameter form 5 to 60 mm are available with the CyberKnife^® ^radiosurgical system. An inverse planning method with non-isocenteric technique was used. The treating physician and physicist input the specific treatment criteria, limiting the maximum dose to critical structures (Figure 1C). The planning software calculated the optimal solution for treatment. The DVH of each plan was evaluated until an acceptable plan was generated. Strict adherence to critical normal structure dose constraints was maintained (Table 1).

### CyberKnife Treatment Planning Parameters

#### Treatment Volume

Treatment volume was defined as the volume contoured on the planning CT scan by the treating neurosurgeon plus a 1 cm margin when clinically indicated. In this study, there was no limit set on the treatable target volumes.

#### Homogeneity Index

The homogeneity index (HI) describes the uniformity of dose within a treated target volume, and is directly calculated from the prescription isodose line chosen to cover the margin of the tumor:

HI = Maximum dose/prescription dose

#### New Conformity Index

The new conformity index (NCI) as formulated by Paddick [21], and modified by Nakamura [22] describes the degree to which the prescribed isodose volume conforms to the shape and size of the target volume. It also takes into account avoidance of surrounding normal tissue.

#### Percent Target Coverage

PTC = The percentage of the target volume covered by the prescription isodose line.

### CyberKnife Treatment Delivery

Image-guided radiosurgery was employed to eliminate the need for stereotactic frame fixation. Using computed tomography planning, target volume locations were related to cranial landmarks. With the assumption that the target position is fixed within the cranium, cranial tracking allows for anatomy based tracking relatively independent of patient's daily setup. Position verification was validated every third beam during treatment using paired, orthogonal, x-ray images [23,24].

### Chemotherapy

Patients received concurrent and/or adjuvant chemotherapy at the discretion of their medical oncologist. Typically, patients were administered Temozolomide with concurrent radiation at a dose of 75 mg/m2/d, given 7 d/wk from the first day of conventional irradiation until the last day of CyberKnife treatment. After a 4-week break, patients generally received 6 cycles or more of adjuvant Temozolomide on a 5-day schedule of 150 to 200 mg per square meter every 28 days.

### Clinical Assessment and Follow-up

Clinical evaluation and MRI imaging were performed at 3-6 month intervals following CyberKnife treatment for 5 years. Evaluation frequency beyond 5 years was determined by the medical oncologist. Throughout the follow-up period, a multidisciplinary team of neurosurgeons, radiation oncologists, medical oncologist and radiologists reviewed outcomes at a weekly central nervous system tumor board. Toxicity was scored according to the National Cancer Institute Common Terminology Criteria for Adverse Events, Version 3.0 [25]

### Statistical Analysis

The follow-up duration was defined as the time from the date of surgery to the last date of follow-up for surviving patients or to the date of death. Actuarial survival and local control was calculated using the Kaplan-Meier method.

## Results

### Patient and Tumor Characteristics

Twenty four consecutive eligible patients were treated over a seven year period extending from January 2002 to January 2009 (Table 2) and were followed for a minimum of 12 months or until death. The mean age of the group was 52 years (range, 27-72). Tumors were evenly distributed between anaplastic glioma (WHO III) and glioblastoma multiformi (WHO IV). Ninety-two percent of the tumors involved the temporal and/or frontal lobes.

### Treatment Characteristics

Thirteen tumors were completely resected; eleven were subtotaly resected. All patients received conventional radiation therapy following tumor resection, with a median dose of 50 Gy (range: 40 - 50.4 Gy). Upon completion of conventional treatment, an additional dose of 10 Gy was delivered in five successive 2 Gy daily fractions utilizing the CyberKnife^® ^image-guided radiosurgical system. Treatment plans were composed of hundreds of pencil beams shaped using a single circular collimator to generate highly conformal plans (mean new conformity index of 1.62, Table 3). Selected plans were inhomogeneous by design (mean homogeneity index of 1.38, Table 3) to minimize dose to adjacent critical structures. Radiation was delivered to a mean prescription isodose line of 73% (Table 3) in 5 approximately 1 hour long treatments. On average, 173 beams were employed to treat the mean prescription volume of 174 cc with a mean percent target coverage of 94%. An average of 58 verification images were taken during each treatment to account for intrafraction patient motion. Twenty-one patients received concurrent and/or adjuvant Temozolmide. Two patients received adjuvant procarbazine, lomustine, vincristine (PCV) alone and one patient declined chemotherapy.

**Table 3:** Treatment Characteristics

Characteristic	
Homogeneity Index	
Min	1.22
Max	1.67
Mean	1.38
Median	1.43

New Conformality Index	
Min	1.20
Max	1.84
Mean	1.62
Median	1.54

Prescription Isodose Line (%)	
Min	60
Max	80
Mean	73
Median	70

Treatment Volume (cc)	
Min	13
Max	550
Mean	174
Median	95

Percent Tumor Coverage	
Min	79
Max	99
Mean	94
Median	96

Number of Radiation Beams Utilized	
Min	87
Max	288
Mean	173
Median	151

Number of Verification Images Per Treatment	
Min	29
Max	96
Mean	58
Median	50

### Outcomes

**Figure 2:**
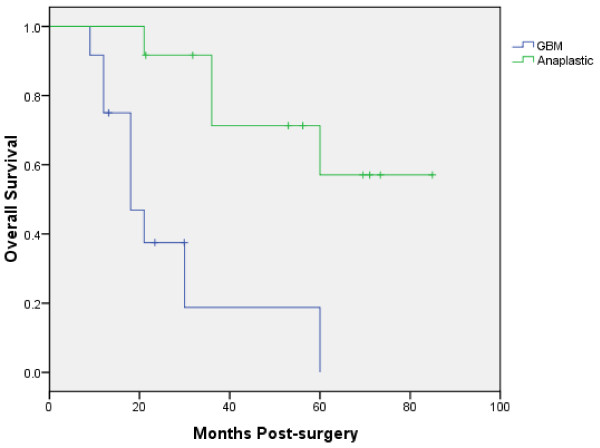
**Kaplan-Meier plot of overall survival**.

The median follow-up was 23 months (range, 13-60 months) for glioblastoma multiforme patients and 63 months (range, 21-85 months) for anaplastic glioma patients (Table 4). No patients were lost to follow-up. Nine of twelve GBM patients (75%) experienced local progression, seven of which died during the follow-up period. Six of the twelve anaplastic patients (50%) experienced local progression, four deaths occurred during the clinical follow-up period. The median time to local progression was 16 months for the glioblastoma multiformi group and 33 months for the anaplastic glioma group. The median survival was 18 months for the glioblastoma multiforme group with a two-year survival rate of 37%. The median survival was not reached for the anaplastic glioma group and the 4-year survival rate was 71% (Figure 2). Of those who died in the glioblastoma multiforme group, 7 (89%) had local disease progression and of those who died in the anaplastic glioma group 4 (100%) had local disease progression (Figure 2). The median time to death was 18 months for the glioblastoma multiformi group and 36 months for the anaplastic glioma group. There were no severe (≥ grade 3) radiation complications per the National Cancer Institute Common Terminology Criteria for Adverse Events, Version 3.0 with this conservative treatment strategy.

**Table 4:** Group Clinical Outcomes

	GBM	Anaplastic
Follow-up (Months)		
Min	13	21
Max	60	85
Mean	22	58
Median	23	63
Time to local progression (Months)		
Min	9	9
Max	60	48
Mean	20	29
Median	16	33
Survival (%)		
2 Year	37	91
4 Year	19	71
Time to Death (Months)		
Min	9	21
Max	60	60
Mean	22	38
Median	18	36
Complications (≥ Grade 3)	0	0

### Salvage Therapy

Ultimately, 16 patients experienced local progression during follow-up (Table 5). Salvage surgery was clinically indicated and pursued in 10 patients, 4 with glioblastoma multiforme and 6 with anaplastic glioma. Each surgery confirmed recurrent glioma with treatment effect. Necrosis was not observed in the absence of tumor progression. Five patients completed salvage chemotherapy, 3 from the glioblastoma multiformi group and 2 from the anaplastic glioma group. A single glioblastoma multiforme patient survived 10 weeks following salvage CyberKnife radiosurgery.

**Table 5:** Individual Clinical Outcomes

Patient	Time to Progression (months)	Vital Status	Time to Death (months)	Clinical Follow-up (months)	Salvage Radiation	Salvage Chemotherapy	Salvage Surgery
1	18	Dead	30	n/a	No	No	No

2	18	Dead	21	n/a	No	No	Yes

3	n/a	Alive	n/a	73	No	No	No

4	36	Dead	36	n/a	No	No	Yes

5	n/a	Alive	n/a	70	No	No	No

6	n/a	Alive	n/a	85	No	No	No

7	n/a	Alive	n/a	71	No	No	No

8	15	Dead	21	n/a	No	No	Yes

9	9	Dead	12	n/a	Yes	No	No

10	30	Dead	36	n/a	No	Yes	Yes

11	48	Dead	60	n/a	No	No	Yes

12	60	Dead	60	n/a	No	Yes	No

13	36	Alive	n/a	56	No	No	Yes

14	9	Dead	12	n/a	No	Yes	Yes

15	n/a	Alive	n/a	53	No	No	No

16	9	Dead	18	n/a	No	No	Yes

17	16	Dead	18	n/a	No	No	No

18	30	Alive	n/a	30	No	No	Yes

19	n/a	Alive	n/a	32	No	No	No

20	12	Dead	18	n/a	No	No	No

21	9	Alive	n/a	21	No	Yes	Yes

22	16	Alive	n/a	23	No	Yes	No

23	n/a	Dead	9	n/a	No	No	No

24	n/a	Alive	n/a	13	No	No	No

## Discussion

High grade gliomas adjacent to critical structures are difficult to treat with conventional radiation therapy technique alone [15]. When irradiating such tumors strict adherence to critical normal structure dose constraints may spare tumors full dose irradiation, potentially resulting in premature local failure and death. Conversely, delivering high doses of radiation immediately adjacent to critical structures without strict limitation increases the risk of late radiation induced complications [9]. Temozolomide-related improvements in high-grade glioma survival have amplified this risk. The number of patients with glioblastoma multiforme surviving past two years is increasing (> 20%) [4] and more than half of patients with anaplastic gliomas are expected to live longer than 4 years. [5,6] These statistics justify current attempts to limit late radiation morbidity. While 3D-conformal radiation therapy [26] and IMRT [27] treatment plans appear to adequately treat the target volume and spare adjacent critical structure, documented set-up inaccuracies and uncorrected intrafraction patient motion increase the risk of potentially costly radiation misadministration.

In this study, we utilized the highly conformal and accurate fractionated CyberKnife radiotherapy to enhance conventional radiotherapy and investigated the safety and efficacy of this technique. The CyberKnife^® ^radiosurgical system has several advantages over conventional radiation delivery systems. Since hundreds of non-isocentric treatment beams are available, the CyberKnife is capable of delivering a highly conformal treatment [17,18]. Cranial tracking, using skeletal anatomy to position the radiation beam, is as precise as frame-based approaches (accuracy <1 mm) [28-31]. Furthermore, by rendering invasive head frames unnecessary, the CyberKnife approach facilitates fractionate treatment while maintaining radiosurgical accuracy.

**Figure 3:**
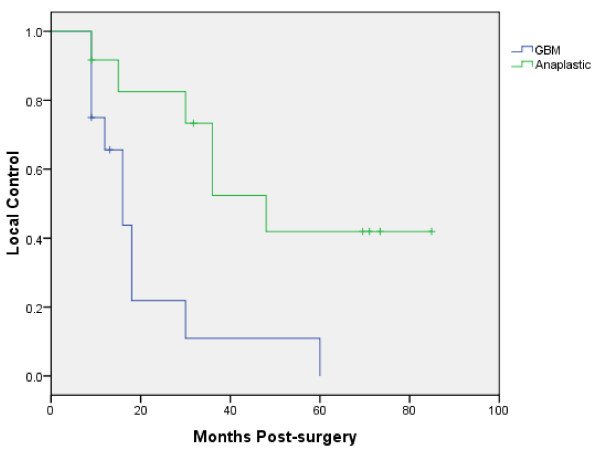
**Kaplan-Meier plot of local control**.

This is the first study to evaluates CyberKnife enhanced conventionally fractionated radiation therapy and chemotherapy for high-grade gliomas. Twenty-four patients were treated with encouraging 2 year and 4 year overall survival rates of 37% and 71% for the glioblastoma multiforme and anaplastic glioma cohorts, respectively. There were no severe late toxicities attributed to this technique using conventional total radiation doses of approximately 60 Gy. Our results demonstrate the feasibility, tolerability and efficacy of delivering CyberKnife enhanced conventionally fractionated radiation therapy and chemotherapy. Unfortunately, local progression remains the predominant pattern of failure for these patients despite optimal radiation treatment and chemotherapy (Figure 3) as confirmed by our salvage surgery analysis (Table 5). Nonetheless, image-guided radiation remains a useful tool to optimize available treatment for patients with tumors in close proximity to critical structures.

## Competing interests

BC is an Accuray clinical consultant.

## Authors' contributions

EO participated in data collection, data analysis and manuscript preparation. BC participated in drafting the manuscript, treatment planning, data collection and data analysis. KE participated in data collection, data analysis and manuscript revision. XY participated in treatment planning, data collection and data analysis. SL participated in treatment planning, data collection and data analysis. SS created tables and figures and participated in data analysis and manuscript revision. HH participated in data collection, data analysis and manuscript revision. JK participated in data collection, data analysis and manuscript revision. HP created tables and figures and participated in data analysis and manuscript revision. AE participated in data collection, data analysis and manuscript revision. CK participated in treatment planning, data analysis and manuscript revision. KM participated in treatment planning, data analysis and manuscript revision. DS participated in data analysis and manuscript revision. WJ participated in treatment planning, data analysis and manuscript revision. SC participated in drafting the manuscript, treatment planning, data collection and data analysis. All authors have read and approved the final manuscript.
